# Catalytic Hydrogen Evolution from H_2_S Cracking over Cr_x_ZnS Catalyst in a Cylindrical Single-Layered Dielectric Barrier Discharge Plasma Reactor

**DOI:** 10.3390/ma15217426

**Published:** 2022-10-23

**Authors:** Saba Afzal, Humaira Hussain, Muhammad Yasin Naz, Shazia Shukrullah, Irshad Ahmad, Muhammad Irfan, Salim Nasar Faraj Mursal, Stanislaw Legutko, Izabela Kruszelnicka, Dobrochna Ginter-Kramarczyk

**Affiliations:** 1 Department of Physics, University of Agriculture Faisalabad, Faisalabad 38040, Pakistan; 2 Department of Chemistry, University of Okara, Okara 56300, Pakistan; 3 Electrical Engineering Department, College of Engineering, Najran University, Najran 61441, Saudi Arabia; 4 Faculty of Mechanical Engineering, Poznan University of Technology, 60-965 Poznan, Poland; 5 Department of Water Supply and Bioeconomy, Faculty of Environmental Engineering and Energy, Poznan University of Technology, 60-965 Poznan, Poland

**Keywords:** Cr-doped ZnS, photocatalysis, hydrogen sulfide, hydrogen, dielectric barrier discharge

## Abstract

The use of non-thermal plasma technology in producing green fuels is a much-appreciated environmentally friendly approach. In this study, an Al_2_O_3_-supported Cr_x_ZnS semiconductor catalyst was tested for hydrogen evolution from hydrogen sulfide (H_2_S) gas by using a single-layered dielectric barrier discharge (DBD) system. The Al_2_O_3_-supported Cr_x_ZnS catalyst (x = 0.20, 0.25, and 0.30) was produced by using a co-impregnation method and characterized for its structural and photocatalytic characteristics. The discharge column of the DBD system was filled with this catalyst and fed with hydrogen sulfide and argon gas. The DBD plasma was sustained with a fixed AC source of 10 kV where plasma produced species and UV radiations activated the catalyst to break H_2_S molecules under ambient conditions. The catalyst (hexagonal-cubic-sphalerite structure) showed an inverse relationship between the band gap and the dopant concentration. The hydrogen evolution decreased with an increase in dopant concentration in the nanocomposite. The Cr_0.20_ZnS catalyst showed excellent photocatalytic activity under the DBD exposure by delivering 100% conversion efficiency of H_2_S into hydrogen. The conversion decreased to 96% and 90% in case of Cr_0.25_ZnS and Cr_0.30_ZnS, respectively.

## 1. Introduction

Hydrogen sulfide (H_2_S) is a poisonous gas and its production is harmful to both human health and equipment [1]. Hydrogen can be produced from various raw materials like coal, water, natural gas, hydrogen sulphide, biomass and boron hydrides using various methods (electrolytic, thermal and photolytic) [2,3]. Currently, the yearly global production of hydrogen is 50 million tons and more than 95% of it is obtained from fossil fuels. The CO_2_ released by fossil fuels contributes to environmental pollution [3]. Hydrogen can also be produced by cracking hydrogen sulfide (H_2_S) over a suitable catalyst. Hydrogen gas is produced through different methods [4,5]. A large amount of H_2_ gas is used in industrial applications, such as the production of chemicals, oils, fats, fuels, and metal reforming [6]. Currently, the Claus method is considered to be an important hydrogen-sulfide-removal technology. This technique is generally not preferred owing to its high working cost and related environmental issues. In the Claus method, hydrogen accumulating in hydrogen sulfide cannot be regained [7]. Various approaches have been proposed for the decomposition of H_2_S to produce hydrogen (H_2_). These methods include the thermo-chemical method, catalytic decomposition, thermal-diffusion photochemical, electrochemical, and plasma [7,8]. In comparative economic analysis, the thermal decomposition and non-thermal plasma (NTP) methods give better results than other methods due to their lower energy cost. At very high temperatures, the decomposition of H_2_S is very low due to the limitation of the thermodynamic equilibrium. The conventional catalysts do not play a better role in converting H_2_S in thermal catalytic decomposition because H_2_S shows high catalytic reactivity with metal species at elevated temperatures [9]. The NTP technique has been suggested as a potential alternative for the direct decomposition of H_2_S into S and H_2_, particularly due to the accomplishment of high-electron energies within a short time. In NTP, various methods have been used to breakdown H_2_S. Such methods include corona, dielectric barrier discharge plasma, microwave, rotating glow, radio frequency discharge, and gliding arc discharge.

A review of the literature shows that many catalyst-hybrid systems have been investigated for the decomposition of H_2_S in DBD plasma with Al_2_O_3_. In addition to Al_2_O_3_-supported Zn_0.4_Cd_0.6_S, ZnS and CdS have also been used for hydrogen production. The ZnS and CdS showed H_2_S conversion corresponding to 90.9% and 97.9%. On the other hand, Zn_0.4_Cd_0.6_S showed 100% catalytic activity for hydrogen production. However, it is a time-consuming catalyst and took 100 h to complete the process. Some other catalysts were also used with Al_2_O_3_ support to produce H_2_, such as Zn_x_Cd_1-x_S, MoS_2_/Al_2_O_3_, La_x_MnO_3,_ and Mn_2_O_3_. The catalytic performance of these catalysts was checked within 50 to 100 h with 100%, 99%, 52%, and 100% H_2_S conversion, respectively [10,11]. A similar activity of H_2_ production was also observed when Zhao et al. [10] used the Al_2_O_3_-supported Cr_x_ZnS semiconductor. They used different molar ratios of Cr/Zn (x = 0.10, 0.15, 0.20 and 0.25) in their investigations. These molar ratios resulted in 81.8%, 87.4%, 100% and 89.7% conversion of H_2_S, respectively [12,13,14].

The Cr-doped ZnS exhibits high-catalytic activity compared to transition metal-doped ZnS. Barnhart et al. [13] reported that Cr is the 21st most common element in the Earth’s crust, with a concentration of 100 ppm. Poornaprakash et al. [14] explained that chromium is an important metal that has an abundant shell structure. Moreover, due to the closed ionic radius of Cr^3+^ (0.63 Å) and Zn^2+^ (0.74 Å), it is easy for Cr^3+^ to substitute Zn^2+^ and penetrate into the host lattice of ZnS. On the other hand, ZnS also acts as a host material with its bulky band gap (3.67 eV). Due to its low toxicity and low cost, it produces different nanostructures in various research applications. In this research, H_2_ gas was produced from Cr-doped ZnS by non-thermal plasma treatment at atmospheric pressure. This method consumed a very small amount of energy at low temperatures when a catalyst was placed in the quartz discharge tube. The catalyst (Cr_x_ZnS) was prepared by the co-impregnation/wet impregnation method with different molar ratios of Cr/Zn (x = 0.20, 0.25, and 0.30). The advantage of this method is that a layer of active matter can easily be prepared on the catalyst surface. Different characterization techniques such as X-ray diffraction (XRD), Ultraviolet-visible (UV-Vis) spectroscopy, Fourier transform infrared spectroscopy (FTIR) and Scanning transmission electron microscopy (STEM) was used to analyze the catalysts. These analyses gave information about the structure, crystal planes, band gap, and light absorbance. The previously reported methods were time-consuming and energy-intensive compared to our work. This study produced reasonably good results in relatively shorter periods. The Cr_0.20_ZnS showed 100% production of H_2_ within 15 h of the process.

## 2. Experimental Part

### 2.1. Chemicals

All the chemicals, including zinc sulfide (ZnS), gamma-aluminum oxide (γ-Al_2_O_3_)_,_ zinc nitrate Zn(NO_3_)_3,_ and chromium nitrate Cr(NO_3_)_3_ were supplied by Merck & Co., Inc. (Rahway, NJ, USA).

### 2.2. Preparation of Photocatalyst

**Figure 1:**
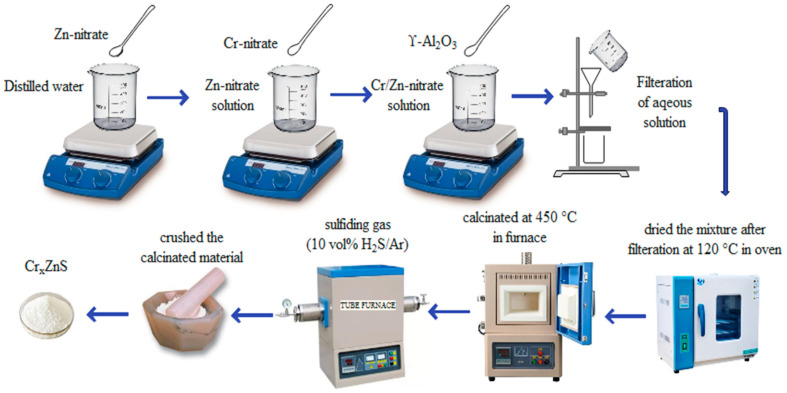
Illustration of the catalyst preparation procedure.

The procedure of synthesis of photocatalyst is illustrated in Figure 1. Using the illustrated procedure, a series of Cr-doped ZnS with Al_2_O_3_ support was prepared with different ratios of chromium (Cr). A wet-impregnation method was adopted to prepare the catalyst samples. In this method, the ZnS amount was taken as 15 g, which is 10 wt% of γ-Al_2_O_3_. An aqueous solution was prepared by adding 5 g of a Zn-nitrate solution to 15 mL of distilled water. The Cr-nitrate and Zn-nitrate were mixed with different molar ratios (0.20, 0.25, and 0.30) by comparing the previous research. The prepared solution and γ-Al_2_O_3_ were mixed with a gentle shake. The mixture was filtered by a filtration process and then dried at 120 °C for 12 h in the oven. The calcination of the material was performed in the furnace for 5 h. A fine powder was formed after crushing the calcinated material. The sulfide catalysts were formed when oxide precursors were sulfidated in the presence of sulfiding gas. Eventually, Cr_x_ZnS catalysts (x= 0.20, 0.25, and 0.30) was prepared. 

### 2.3. DBD Plasma-Assisted Hydrogen Evolution

**Figure 2:**
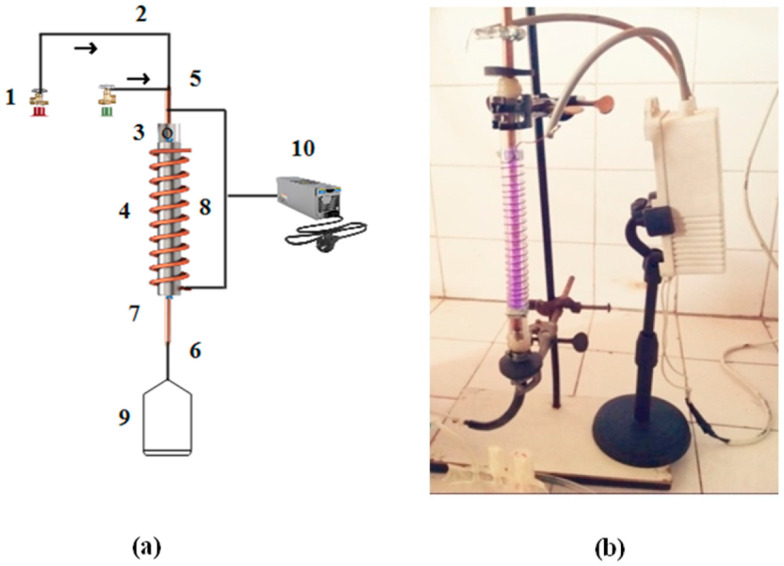
(**a**) Schematic of DBD plasma system. (1) Gas cylinder, (2) Gas supply, (3) Hole in rod, (4) Quartz tube, (5) Inlet, (6) Outlet, (7) Inner electrode, (8) Copper coil, (9) Gas sampling bag, and (10) AC power supply. The arrows indicate the direction of the gas flow (H_2_S and Ar). (**b**) Photographic view of single-layered DBD plasma system for H_2_S decomposition.

The schematic and photographic views of the DBD setup, used for the production of hydrogen by cracking H_2_S molecules over the composite catalyst, are given in Figure 2. This laboratory-built system consists of a 30 cm DBD vertical column with an active plasma column length of 23 cm. A quartz tube with a 4 mm wall thickness and a 12 mm internal diameter was used as a DBD column. A copper rod of 8 mm diameter was passed through the tube to work as one of the two electrodes. The tube was wrapped with a copper wire to work as an electrode for uniform radial and spatial distribution of the applied power and plasma. The upper end of the tube was used as a gas inlet and the lower end was connected with the gas analyzer. The discharge column of the DBD system was filled with this catalyst and fed with hydrogen-sulfide and argon gas.

The DBD plasma was sustained with a fixed AC source of 10 kV where plasma-produced species and UV radiations were used to activate the catalyst to break the H_2_S molecules under ambient conditions. The Al_2_O_3_-supported Cr_x_ZnS semiconductor catalyst was tested for hydrogen evolution from H_2_S gas using this single-layered DBD system [15]. The discharge volume of the dielectric-barrier-discharge reactor was 22 mL [16]. One end of the battery was attached to the wire and the other to the rod. About 10 g of the Cr_x_ZnS catalyst (x = 0.20, 0.25, and 0.30) was loaded in the discharge column. At the same time, the gas (Ar + H_2_S) was passed through the loaded column. The gas product of the reaction in the discharge column was analyzed. The relationship between the H_2_S (X_Hydrogen sulfide_) and H_2_ yield (X_Hydrogen_) is shown as follows:

(1) $$X_{\mathrm{Hydrogen}\;\mathrm{sulfide}}=X_{\mathrm{Hydrogen}}+\frac{A\prime }{A_{o}}\times 100\%$$

where A is the value of the H_2_ peak area of effluence. A_o_ has represented the hydrogen peak area at 100% hydrogen sulfide conversion. The area of the represents the energy lost during a single voltage cycle in the discharge. The total input energy used in the plasma during the process was calculated by the specific input energy (SIE) as:

(2) $$\mathrm{SIE}=\frac{P}{V}$$

where V is the flowrate of gas (L/s) and P is the discharge power (W). The energy utilization for the H_2_ generation (E, eV) was calculated from the specific input energy as:

(3) $$\mathrm{Energy}\;\mathrm{consumption}\;\left(E,\;\mathrm{eV}\right)=\frac{P_{\mathrm{discharge}}}{H_{2}S\;\mathrm{converted}\;\left(\mathrm{mol}/s\right)}\times \frac{1}{96}$$

## 3. Results and Discussion

### 3.1. FTIR Analysis of Catalyst

**Figure 3:**
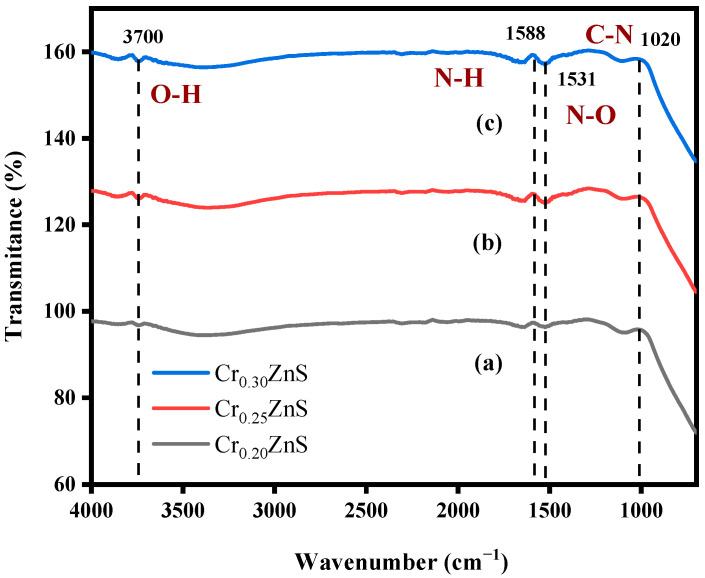
FTIR spectra of (a) Cr_0.20_ZnS, (b) Cr_0.25_ZnS, and (c) Cr_0.30_ZnS catalyst samples.

With the Fourier transform infrared (FTIR) analysis, the absorbance of the species in the crystal surface and the nanoparticle formation of ZnS were checked. It is reported that this analysis also gives information about the chemical bonding of the chemical [17]. The FTIR absorbance spectra of Cr_x_ZnS with different molar ratios are shown in Figure 3. As shown in Table 1, FTIR analysis showed the same peaks for Cr_x_ZnS samples with different ratios (x = 0.20, 0.25, and 0.30) within the range of 500–4000 cm^−1^. The FTIR peaks were located around 3700 cm^−1^, 1588 cm^−1^, 1531 cm^−1^, and 1020 cm^−1^. All the peaks exist in the group frequency region (GFR) except 1020 cm^−1^ because its range was lower than the other three peaks, so it was observed in the fingerprint region (FPR) [18]. The peak at 3700 cm^−1^ was due to O―H stretching vibration. This peak shows an alcohol group of compounds with intermolecular forces based on their structure [19]. The peaks at 1588 cm^−1^ and 1020 cm^−1^ exhibited the same amines groups with no intermolecular force at medium peaks. Both peaks have different vibrations, i.e., 1588 cm^−1^ represents the N―H bending due to GFR and 1020 cm^−1^ represents the C―N stretching vibration in FPR. There is a strong peak appearance at 1531 cm^−1^ caused by N―O stretching. It exists in a nitro-compound group with no bonding forces.

### 3.2. UV-Visible Analysis

**Figure 4:**
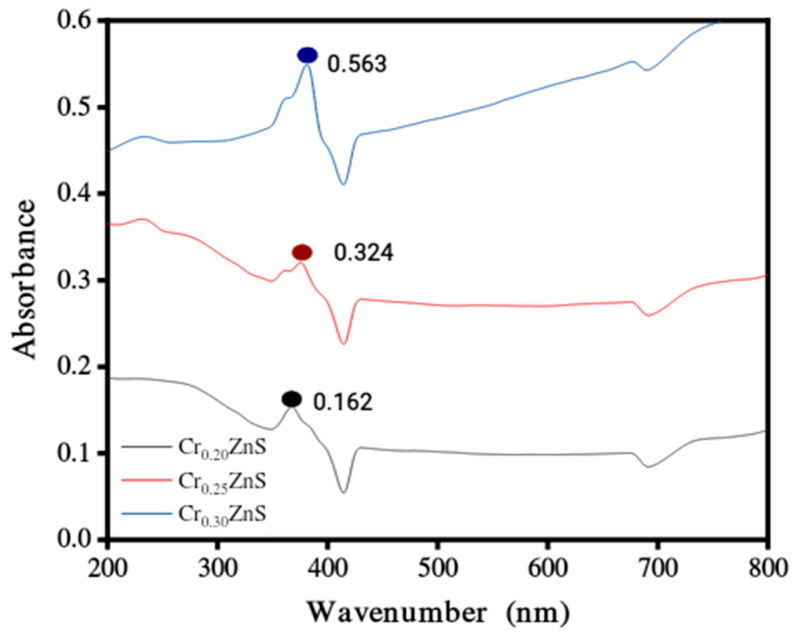
UV-Vis absorbance spectra of Cr_x_ZnS (x = 0.20, 0.25, and 0.30) catalyst.

**Figure 5:**
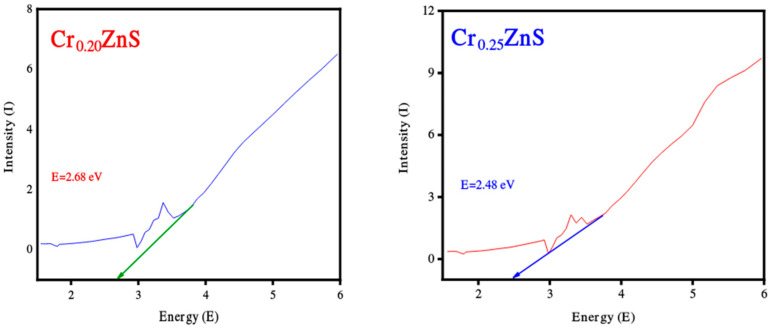

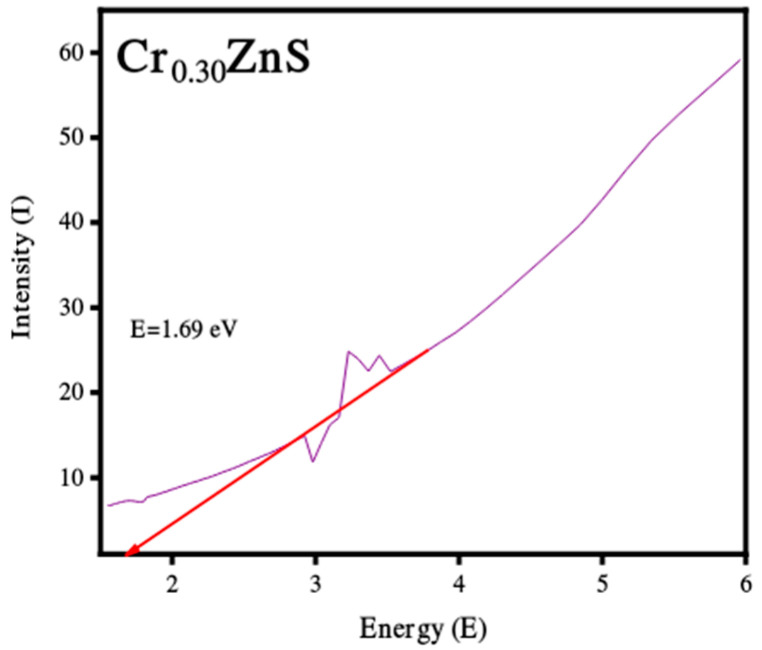
Band gap estimation of Cr_0.20_ZnS, Cr_0.25_ZnS, and Cr_0.30_ZnS catalyst samples.

The absorption spectra of catalysts Cr_x_ZnS (x = 0.20, 0.25, and 0.30) were examined by UV-Vis analysis within the wavelength range of 200 nm to 800 nm and obtained results are shown in Figure 4. The absorption edges at 367 nm, 376 nm, and 379 nm correspond to Cr_0.20_ZnS, Cr_0.25_ZnS, and Cr_0.30_ZnS respectively observed along the x-axis [20]. In Figure 5, the Cr_0.30_ ZnS catalyst showed a superior shift in absorption edge (red-shift) towards the visible light region in contrast to other samples, showing a maximum absorption upto 379 nm [21,22]. Cr_0.20_ZnS, Cr_0.25_ZnS and Cr_0.30_ZnS catalysts represented the absorbance values of 0.162 nm, 0.324 nm and 0.563 nm, respectively. Bodke et al. [14] reported that the concentration of doped Cr^3+^ had a pronounced effect on the optical properties of the ZnS catalyst and witnessed a significant red-shift in the absorption of Cr-doped ZnS.

The band gap values of the catalysts with different Cr compositions are reported in Figure 5. The band gap of Cr_x_ZnS with x = 0.20, 0.25, and 0.30 was found to be 2.68, 2.48, and 1.69 eV, respectively. These band gap values are lower than the standard value of bulk ZnS (3.6 eV) [23].

### 3.3. X-ray Diffraction Analysis

**Figure 6:**
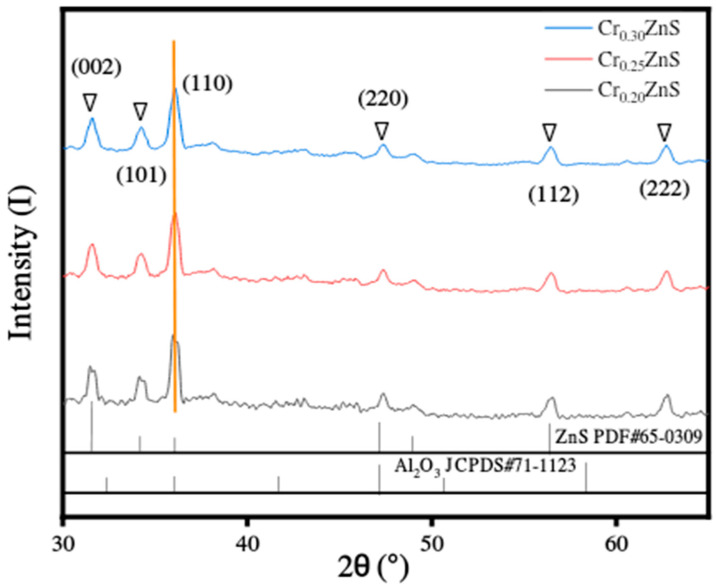
XRD spectra of the Cr_x_ZnS (x = 0.20, 0.25, and 0.30) catalyst.

The XRD analysis of as-synthesized catalysts Cr_x_ZnS (x = 0.20, 0.25, and 0.30) are shown in Figure 6. All prepared samples showed similar diffraction peaks, identifying no variation in the host crystal structure after introducing Cr^3+^ ions into its lattice. The different diffraction peaks were found at 2θ values of 31°, 36°, 47°, and 56°, which correspond to (002), (001), (110), and (112) planes of ZnS, respectively. There was no other obvious indication of any other diffraction peak found except for the alumina peak. Among all samples, only the Cr_0.20_ZnS catalyst showed the origination of diffraction peak related to Cr impurity [24]. The information about the existence of the characteristic peak of (110) plane was confirmed from JCPDS#65-0309. The crystal structure of the Cr_x_ZnS catalyst is cubic sphalerite.

The surface area decreased with an increase in a molar ratio of Cr/Zn. Ramasamy [25] reported that the lattice constants were reduced with Cr doping because of the ionic radius (0.63 Å and 0.74 Å) of Cr^3+^ and Zn^2+^ ions. In our study, as the Cr^3+^ content increased, the lattice parameters were decreased in the case of all as-prepared Cr_x_ZnS catalysts. The Scherrer equation was used to calculate the average crystallite size of the catalyst. The grain sizes of Cr_x_ZnS were estimated to be 18.30, 17.89, and 17.49 nm, corresponding to the Cr_0.20_ZnS, Cr_0.25_ZnS, and Cr_0.30_ZnS, respectively. Since the band gap and the grain size are inversely related to each other; therefore, our measured band gap and crystallite size are in good agreement, as illustrated in Table 2 [26].

### 3.4. STEM Morphology Analysis

**Figure 7:**
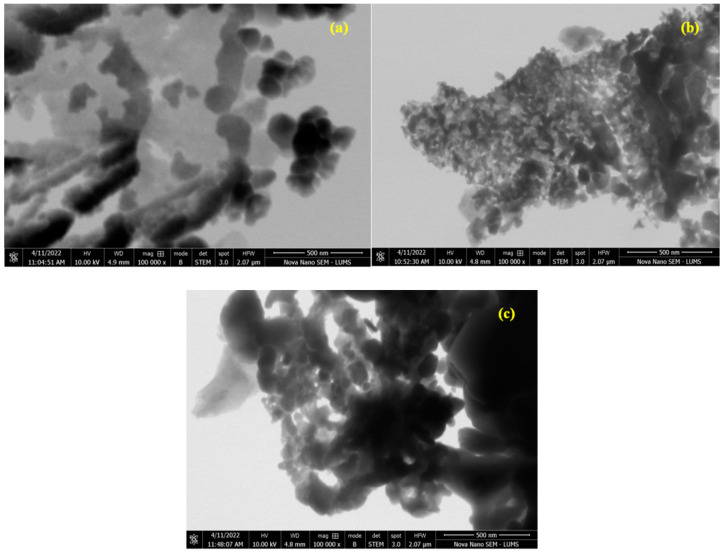
STEM images of (**a**) Cr_0.20_ZnS, (**b**) Cr_0.25_ZnS, and (**c**) Cr_0.30_ZnS catalyst samples.

The morphology of the as-prepared samples was analyzed using the STEM technique and the results are displayed in Figure 7. The STEM analysis confirmed the successful formation of nanoparticles. The fine doping of the catalyst at a ratio of x = 0.30 appeared as a dark area in the images. A rough spherical morphology of the particles was observed in STEM images [27].

**Figure 8:**
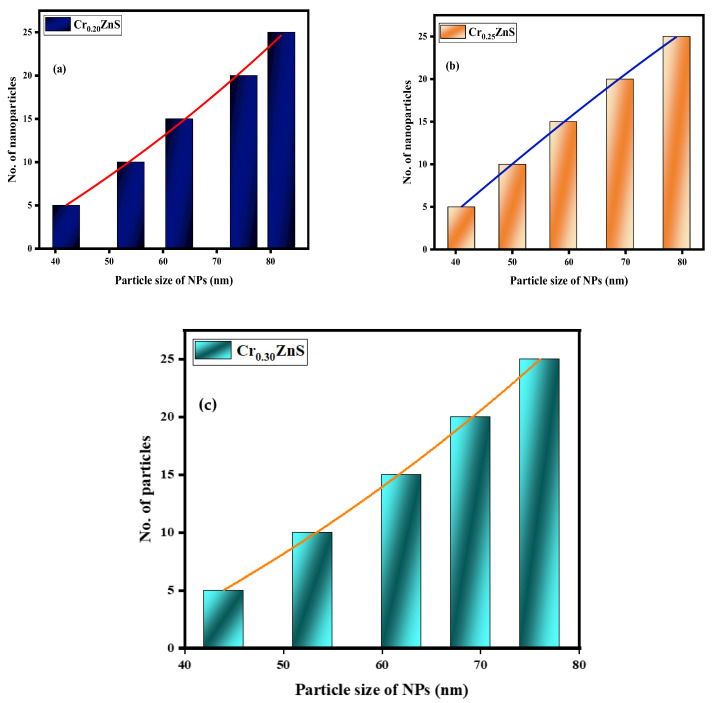
Particle size distribution of (**a**) Cr_0.20_ZnS, (**b**) Cr_0.25_ZnS, and (**c**) Cr_0.30_ZnS catalyst samples.

The statistical distribution of Cr_x_ZnS (x = 0.20, 0.25, and 0.30) is expressed within the range of 1–10 nm [28]. Figure 8 shows the distribution of particle sizes measured from the STEM images. The average particle size of Cr_0.20_ZnS, Cr_0.25_ZnS, and Cr_0.30_ZnS was measured at about 82 nm, 79 nm, and 76 nm, respectively.

### 3.5. Photoluminescence Analysis

**Figure 9:**
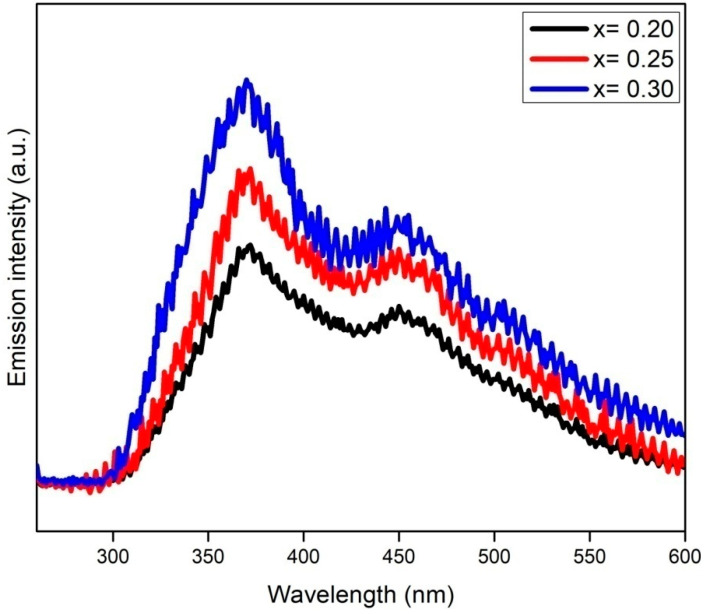
PL spectra of the Cr_x_ZnS (x = 0.20, 0.25, and 0.30) catalyst.

The catalysts were further characterized with PL technique to determine the extent of the photoinduced electron-hole recombination rate. Principally, high-PL-emission intensity represents the rapid recombination of charge carriers and vice versa [29]. Figure 9 shows the PL emission spectra of Cr_x_ZnS catalyst samples measured at room temperature and an exciton wavelength of 325 nm. The Cr doping has successfully altered the surface of the ZnS and promoted the migration of surface carriers, causing an increment in light-harvesting, which is consistent with the UV-Vis results [30]. The Cr_x_ZnS (x = 0.20) catalyst demonstrated the lowest emission intensity compared to the other two catalysts, identifying its effective suppression of charge carriers. It is worth mentioning that the PL intensity was reduced with Cr doping in the UV and visible zone because of the effective role of Cr^3+^ ions in trapping the electrons to prolong their recombination with holes [31]. Additionally, Cr^3+^ dopants provide electrons reaching the surface of the ZnS to effectively initiate the reaction to accelerate the photocatalytic process [32]. Hence, it is concluded that the PL intensity is reduced owing to a strongly inhibited recombination of photoinduced charge carriers because Cr^3+^ captured the electrons. The Cr_x_ZnS (x = 0.20) catalyst demonstrated the least intensity; therefore, it is more appropriate for hydrogen production.

### 3.6. Hydrogen Evolution Activity

**Figure 10:**
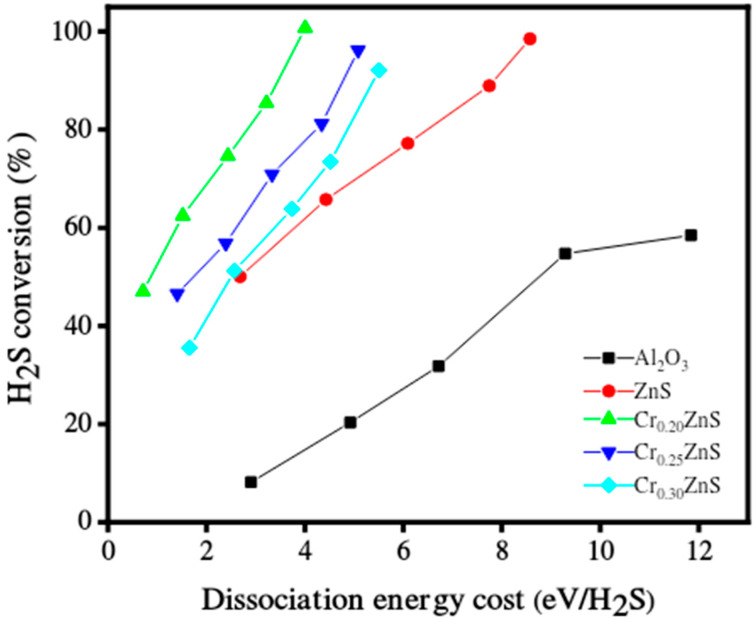
Hydrogen sulfide decomposition over the catalyst in DBD plasma environment.

The catalytic performance of the Cr_x_ZnS catalyst samples was evaluated for hydrogen production under non-thermal plasma treatment. The catalytic performance of un-doped ZnS and Al_2_O_3_ as a support material was also presented. The decomposition of H_2_S over the tested catalyst compositions in a single-layered DBD plasma environment is reported in Figure 10. In the case of Al_2_O_3_ support, both discharge diffusion and plasma-produced reactive species may be influenced. The residence time of these species may be extended by the adsorption capacity of the Al_2_O_3_ support [33]; however, in the literature, the electric field was enhanced by using porous materials. Both the discharge and prolonged residence time are useful for H_2_S decomposition [34]. More micro-discharges occurred in the Al_2_O_3_-filled gap, which led to the beginning of chemical processes involving H_2_S molecules, radicals, and electrons. All prepared Cr_x_ZnS catalysts showed better performance of H_2_S conversion than that of pure ZnS and Al_2_O_3_ support. The Cr_x_ZnS catalyst with a molar ratio of x = 0.20 showed the highest decomposition of H_2_S.

The results after comparison revealed that H_2_S conversion varied for different Cr/Zn molar ratios. The catalytic activity greatly depends upon the dopant concentration. The H_2_S conversion levels significantly impact the energy needed to break down its molecules [35]. The Cr_0.20_ZnS catalyst outperformed the other tested catalysts in terms of catalytic performance and fully converted H_2_S at significantly lower energies. The H_2_S decomposition was 100%, 96%, and 90% when the gap was filled with Cr_0.20_ZnS, Cr_0.25_ZnS, and Cr_0.30_ZnS, respectively. The characterization of the catalyst showed that physical and chemical properties changed with Cr/Zn molar ratio. The cubic sphalerite structure of the catalyst was shown by XRD analysis [36,37]. Cr^3+^ ions of chromium revealed uniformly scattering over the ZnS without introducing separated impurity phases.

**Figure 11:**
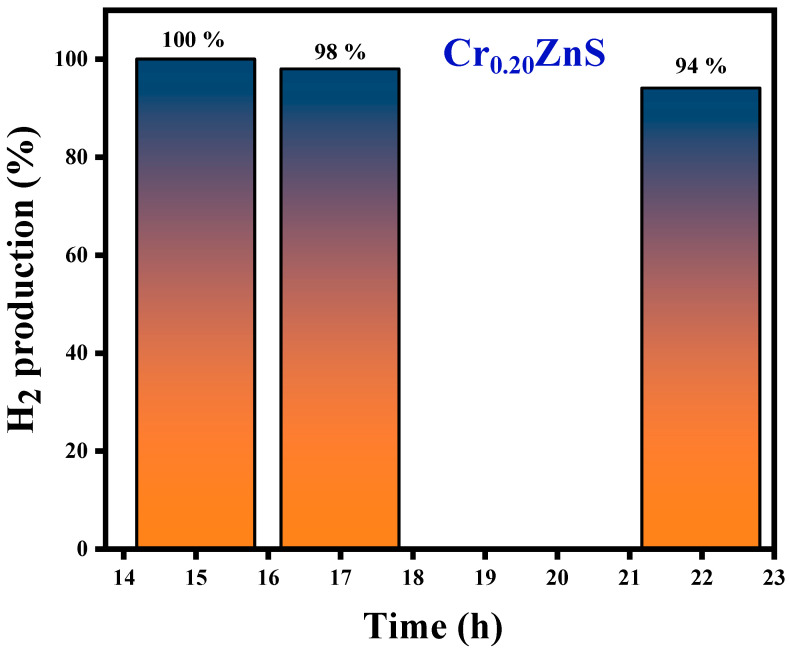
H_2_ production of the Cr_0.20_ZnS catalyst with time.

The H_2_S conversion with specific input energy varies for different H_2_S concentrations over the Cr_0.20_ZnS catalyst. The conversion rate was higher at the lower H_2_S concentrations. H_2_S decomposition increased with increasing the specific input energy. Chivers and Lau [36] showed similar results for the H_2_S conversion under non-thermal plasma conditions. When a large number of electrons collide with Ar balance gas at lower H_2_S concentrations, air balance gas is also crucial to the breakdown. Cr_0.20_ZnS was selected to evaluate the stability of the catalytic after 100% decomposition of H_2_S [38]. The long-term H_2_S conversion reaction of the Cr_0.20_ZnS catalyst is shown in Figure 11. The H_2_ evaluation shows a maximum value up to 15 h and thereafter starts to decrease over time. Three different readings were noted at different time periods. The H_2_ evolution decreased from 100% to 94% over the Cr_0.20_ZnS after 22 h of reaction time. A decrease in H_2_ production over time might be due to the deactivation of the catalyst.

Table 3 summarizes the findings of hydrogen production efficiency over the Cr_x_ZnS catalyst samples. The H_2_ production during the conversion of H_2_S was 100%, 96%, and 90% for x = 0.20, 0.25, and 0.30, respectively. Different energy conversion was observed with the same SIE (specific input energy) values for all catalysts.

## 4. Conclusions

This laboratory-built non-thermal plasma system with a vertical DBD column was used to decompose H_2_S over the Cr_x_ZnS catalyst for the production of hydrogen gas. The catalyst was prepared using the co-impregnation method. A FTIR spectrum showed the materials’ absorbance in different regions (fingerprint and group frequency region) and functional groups. X-ray diffraction displayed the surface morphology of the catalyst. The values of intensity, millar indices, grain size, and d-spacing were decreased with increasing the Cr concentration. Hydrogen evolution was maximized (100%) after 15 h of reaction over the Cr_0.20_ZnS. Hydrogen evolution then decreased to 94% after 22 h of reaction time, showing a decrease in catalytic activity over time. The Cr_0.20_ZnS, Cr_0.25_ZnS, and Cr_0.30_ZnS catalysts showed 100%, 96%, and 90% conversion, respectively, after 15 h of processing time. The earlier reported works are time-consuming and energy-intensive compared to our work. This study produced reasonably good results in relatively shorter periods. The Cr_0.20_ZnS showed 100% conversion of H_2_S within 15 h of the process.

## Figures and Tables

**Table 1:** FTIR peaks and corresponding groups of the Cr_x_ZnS catalyst.

FTIR peaks	Spectrumregion	Appearance	Bonding force	Group	Compoundclass
3700	GFR	medium, sharp	intermolecular	O―H stretching	alcohol
1588	GFR	medium	-	N―H bending	amines
1531	GFR	strong	-	N―O stretching	nitro- compound
1020	FPR	medium	-	C―N stretching	amines

**Table 2:** The band gap and grain size of Cr_x_ZnS catalyst samples.

Catalyst	FWHM	Grain size (nm)	Band gap (eV)
Cr_0.20_ZnS	0.477	18.30	2.68
Cr_0.25_ZnS	0.488	17.89	2.48
Cr_0.30_ZnS	0.499	17.49	1.69

**Table 3:** Conversion efficiency, specific input energy, and energy consumption for catalytic hydrogen production.

Catalyst	H_2_S conversion (%)	SIE (J/L)	Energy consumption (eV)
Cr_0.20_ZnS	100	14.66	0.120
Cr_0.25_ZnS	96	14.66	0.124
Cr_0.30_ZnS	90	14.66	0.138

## Data Availability

Data is available from the authors on reasonable request.
